# Concentrations of Essential Trace Metals in the Brain of Animal Species—A Comparative Study

**DOI:** 10.3390/brainsci10070460

**Published:** 2020-07-17

**Authors:** Chiara Alessia DeBenedictis, Andrea Raab, Ellen Ducie, Shauna Howley, Joerg Feldmann, Andreas Martin Grabrucker

**Affiliations:** 1 Cellular Neurobiology and Neuro-Nanotechnology Lab, Department of Biological Sciences, University of Limerick, V94PH61 Limerick, Ireland; Chiara.DeBenedictis@ul.ie (C.A.D.); ellentducie@gmail.com (E.D.); shaunahowley@gmail.com (S.H.); 2 Bernal Institute, University of Limerick, V94T9PX Limerick, Ireland; 3 Trace Element Speciation Laboratory (TESLA), Department of Chemistry, University of Aberdeen, Aberdeen AB24 3UE, UK; a.raab@abdn.ac.uk (A.R.); j.feldmann@abdn.ac.uk (J.F.); 4 Institute of Chemistry, University of Graz, A-8010 Graz, Austria; 5 Institute of Chemistry, Environmental Analytical Chemistry, University of Graz, 8010 Graz, Austria; 6 Health Research Institute (HRI), University of Limerick, V94T9PX Limerick, Ireland

**Keywords:** zinc, iron, copper, selenium, ICP-MS, CNS, locust, earthworm, herring, biometals, pig, mouse, rat, snail

## Abstract

The essential trace metals iron, zinc, and copper have a significant physiological role in healthy brain development and function. Especially zinc is important for neurogenesis, synaptogenesis, synaptic transmission and plasticity, and neurite outgrowth. Given the key role of trace metals in many cellular processes, it is important to maintain adequate levels in the brain. However, the physiological concentration of trace metals, and in particular zinc, in the human and animal brain is not well described so far. For example, little is known about the trace metal content of the brain of animals outside the class of mammals. Here, we report the concentration of iron, zinc, and copper in fresh brain tissue of different model-species of the phyla Chordata (vertebrates (mammals, fish)), Annelida, Arthropoda (insects), and Mollusca (snails), using inductively coupled plasma mass-spectrometry (ICP-MS). Our results show that the trace metals are present in the nervous system of all species and that significant differences can be detected between species of different phyla. We further show that a region-specific distribution of metals within the nervous system already exists in earthworms, hinting at a tightly controlled metal distribution. In line with this, the trace metal content of the brain of different species does not simply correlate with brain size. We conclude that although the functional consequences of the controlled metal homeostasis within the brain of many species remains elusive, trace metal biology may not only play an important role in the nervous system of mammals but across the whole animal kingdom.

## 1. Introduction

The human body is in need of the essential trace metals iron, zinc, copper, manganese, cobalt, and molybdenum [1,2]. The status of chromium is still debated. Of these, cobalt occurs mostly as cobalamin (Vitamin B12) and molybdenum is only needed for three enzymes as far as known [3,4,5,6,7,8]. Thus, cobalt, molybdenum, and also manganese, occur at very low concentrations.

Within the brain, iron is the trace metal with the highest concentration, followed by zinc and copper [9,10,11,12]. However, most iron is contributed by heme-bound iron in hemoglobin of red blood cells found within the vast capillary network of the brain. Several studies have shown that these trace metals are involved in a plethora of biological processes. For example, iron acts as a cofactor for different enzymes involved in neurotransmitter synthesis such as tryptophan hydroxylase and tyrosine hydroxylase [13,14,15]. In addition to this, iron plays a pivotal role in the proper myelination of neuronal axons in the central nervous system (CNS) [16,17], in synaptic plasticity [18], and in regulating the neuronal energy status [19]. Studies in animal models underline the critical role of iron in neurodevelopment. Iron deficiency in the early stages of life results not only in acute brain dysfunction during the iron deficiency, but also in long-lasting abnormalities even after iron repletion [20]. 

The second most abundant trace metal in brain, zinc, is involved in several functions [11]. Zinc is a structural and a catalytic component of proteins, it is a cofactor for over 300 enzymes and metalloproteins, it provides stability to a series of transcription factors, it assumes neuroprotective properties, and it is also involved in the defense of the body and brain against oxidative stress [11,21,22,23]. Zinc in the brain has key roles in synaptic plasticity, in the regulation of neurogenesis, neuronal migration and differentiation, and in the modulation of neurotransmission [24,25,26,27], thereby mediating healthy cognitive development and brain functioning [28]. 

Copper is the third most abundant trace metal within the brain [12]. Copper in the brain is a co-factor for essential enzymes mediating, among others, neurotransmitter biosynthesis, and the oxidative stress response [29,30]. Therefore, copper homeostasis is essential for a healthy CNS development and function. For example, Menkes disease, characterized by a copper deficiency in brain tissue, is associated with demyelination and neurodegeneration [31,32].

Intriguingly, abnormal metal levels during brain development such as zinc deficiency and copper overload have been identified as risk factors for autism spectrum disorders (ASD), highlighting the importance of intact metal homeostasis for healthy brain development [33,34,35]. However, the literature shows conflicting information as to how much iron, zinc, and copper should be regarded as the physiological concentration in the brain of humans and their primary animal models for biomedical research, mice and rats. Moreover, hardly anything is known about the trace metal concentration of animals of other phyla of the animal kingdom, such as the brain (ganglions) of Annelida, Mollusca, or Arthropoda. Even within our own phylum (Chordata) and subphylum, the Vertebrata, apart from mouse and rat, few studies investigated the levels of trace metals in the brain of reptiles, amphibians, fish, or birds. Therefore, almost all our information about the molecular function of trace metals, and in particular zinc, is based on studies on rodents. However, the abundance and role of zinc in the brain of mollusks, for example, is largely unknown. Some studies published between 1984 and 2019 have reported concentrations of zinc in brain tissue (Table 1). 

However, from these studies, it is obvious that insights are limited to very few species. In addition, a comparison of the results from the studies reveals sometimes large differences in the detected levels of trace metals in the brain. These differences may be based on different methods for tissue preparation, a not standardized protocol for the measurements, and the use of animals/participants with different gender, age, or diet. For example, while one study found a zinc concentration of 32.93 ± 0.12 mg/kg in the cerebellum of Wistar rats [36], the same brain region was reported to have 15.00 ± 5.50 mg/kg in a separate study [37].

Given that restoring or manipulating trace metal levels in the brain is an emerging treatment strategy, for example in Alzheimer’s disease [51,52] and ASD [53,54,55], the lack of information is affecting research as it is unclear, what may be considered a physiological concentration of a specific metal in the brain. In addition, it is not well investigated whether the trace metal concentration of model organisms for human research is comparable to humans, and comparable between the models, e.g., mouse and rats. Moreover, almost nothing is known about trace metal levels in species of other phyla, and therefore, it is difficult to assess whether the brains of vertebrates, or mammals in particular, are special in terms of their trace metal content.

To close this knowledge gap, we have chosen a set of animals that represent different phyla within the animal kingdom such as earthworms (Annelida), locusts (Arthropoda), snails (Mollusca), and several animals of our phylum (Chordata) such as the model organisms mice, rats, but also pig and a non-mammalian vertebrate (fish). We investigated whether tissue preparation protocols have influence on the results and measured iron, zinc, and copper concentrations across all animals following the same protocol and method. In addition, we included the essential non-metal selenium in our measurements [56]. We investigated whether significant differences in trace metal levels are visible in the representatives of the phyla and whether this follows a pattern e.g., correlates with an increase in brain size or organization. Finally, we compared our results with the average trace metal concentrations found in our comparative analysis of the existing literature to validate the existing findings.

## 2. Materials and Methods

### 2.1. Materials

*Lumbricus terrestris* and *Schistocerca gregaria* were obtained from Newlands Home and Garden Center, Dublin (Ireland). *Helix lucorum* were obtained from Sabarto (France). *Clupea harengus* was obtained from Rene Cusack Fish, Limerick (Ireland), *Mus musculus* and *Rattus norvegicus* were obtained from Janvier labs (France) for the preparation of snap frozen brain tissue. Perfusion fixed *Rattus norvegicus* were obtained from Carolina Biologicals (USA). All mouse/rat experiments were performed in compliance with the guidelines for the welfare of experimental animals issued by the Federal Government of Germany and approved by the Regierungspräsidium Tübingen and the local ethics committee (Ulm University) (project code-O.103). *Mus musculus* and *Rattus norvegicus* received a standard laboratory diet (ssniff GmbH, Germany). *Sus scrofa domesticus* was sourced from a local abattoir (Nenagh, Ireland), approved by the ethics committee of the Faculty for Science and Engineering, University of Limerick, Ireland. Unless otherwise indicated, all chemicals were obtained from Sigma-Aldrich/Merck (Ireland). PTFE-coated tweezers were used for dissection.

### 2.2. Preparation of Brain Tissues

Earthworm—(Annelida: *Lumbricus terrestris* (Common earthworm)) Live earthworms (50–75 mm) were obtained and killed in a 2:1 water/chloroform solution before dissection. Each earthworm was washed with 70% ethanol to remove soil and debris. An incision was made using a scissors at the tail end of the earthworm and along the length of the earthworm. The brain/head ganglion was removed using a tweezers. In order to dissect the ventral nerve cord, the intestine was removed, and the nerve cord extracted. Brain and nerve cord were transferred to separate endotoxin free sterile plastic tubes and snap frozen in liquid nitrogen.

Locust—(Arthropoda: Insecta: *Schistocerca gregaria* (Desert locust)) Live locust hoppers (36–42 mm) were killed by freezing at −20 °C. The legs and antenna were removed, and an incision was made between the eyes of the locust. The exoskeleton of the head was removed to allow for direct access to the brain. Using a dissection-microscope the locust brain that is comprised of three pairs of ganglia that lie above the esophagus was taken out using tweezers and transferred to an endotoxin free sterile plastic tube and snap frozen in liquid nitrogen.

*Snail*—(Mollusca: Gastropoda: *Helix lucorum* (Turkish snail)) Snails were killed and stored without shell in water. An incision was made between the antenna of the snail down to the base of the snail’s head avoiding all internal organs/structures, and its skin was subsequently removed. A dissection microscope was used to view the snail head. The brain was located and extracted using tweezers. Once removed these samples were transferred to an endotoxin-free sterile plastic tube and snap frozen in liquid nitrogen.

Fish—(Chordata: *Clupea harengus* (Atlantic herring)) The fish was obtained fresh frozen. Prior to dissection, the herring were thawed, and the head was removed using a razor blade. The cranial bone from the ventral and the dorsal side of the brain was removed and the brain taken out using tweezers and transferred to an endotoxin free sterile plastic tube and snap frozen in liquid nitrogen.

Mouse—(Chordata: Mammalia: *Mus musculus C57BL/6*) and *Rat*—(Chordata: Mammalia: *Rattus norvegicus domestica* (*Wistar*)) Mice and rats were killed using CO_2_. The heads were removed, and an incision was made between the eyes and across the skull of the rat. The tip of the forceps was used beneath the brain stem region to ease out the brain with minimal disruption. Subsequently, the brain regions striatum, cortex, thalamus, hippocampus, and cerebellum were removed and transferred to an endotoxin free sterile plastic tube and snap frozen in liquid nitrogen.

Pig—(Chordata: Mammalia: *Sus scrofa domesticus* (domestic pig)) The fresh pig’s head was removed from its body prior to the brain dissection. First, the ears and skin were removed, and a saw was used to make horizontal incisions. A hammer and chisel were used to allow for further incisions to be made deeper into the bone of the skull. Following this, the skull was opened, and the brain extracted from the skull. The meninges were removed using tweezers. Any remaining membrane sections were removed using the forceps. Using a scalpel, tissue from the prefrontal cortex was removed and transferred to an endotoxin free sterile plastic tube and snap frozen in liquid nitrogen.

#### 2.2.1. Preparation of Brain Homogenate

Buffer A Lysis buffer (0.12 g Hepes and 5.48 g Sucrose/50 mL ddH_2_O, pH 7.5) was added to the tissue (10 mL/g tissue) prior to homogenization on ice using a Sonicator (Fisher Scientific, Waltham, MA, USA). After homogenization of the whole brain or brain region, a Proteinase K digestion was performed with 1 mL of crude homogenate and 8 Μl proteinase K for 1 h at 37 °C. Following the incubation, the samples were centrifuged for 10 min at 3200 rpm and the supernatant was collect for the ICP-MS measurements.

#### 2.2.2. ICP-MS Measurement

*ICP-MS* analysis was carried out by TELSA—Trace Element Speciation Laboratory, Department of Chemistry, in the University of Aberdeen, UK. A total of 10 μL yttrium solution was added as an internal standard. Following this, digestion of the samples was carried out using 50 μL nitric acid on a shaking incubator at 90 °C for 30 min. Following cooling, the samples were adjusted to 1 g using 18 MΩ cm water. Seronorm Trace elements Whole blood L-1^®^ (Table S1a) and Seronorm Trace elements Serum L-1^®^ (Table S1b) were used as certified reference material (CRM). CRMs with added yttrium solution as internal standard were digested using nitric acid (70%) and diluted in the exact same way as the brain samples. Inorganic Ventures 71A standard element mix was diluted with yttrium solution as internal standard to appropriate concentrations using 5% (*v*/*v*) nitric acid for use as external calibration. ICP-MS/MS measurement (Agilent 8800, Cheadle, UK) was carried out using hydrogen (4 mL H_2_/min) as reaction gas in MS/MS mode to remove interferences. An AS110 (CETAC, Cumbernauld, UK) with micronebulizer (50 μL/min) was used for sample introduction. It was ensured that optimization for sensitivity and resolution of the system was carried out before the measurement. All samples were measured with ten replicates using ^89^Y as the internal standard. Isotopes measured were ^56^Fe, ^57^Fe, ^63^Cu, ^65^Cu, ^66^Zn, ^67^Zn, ^68^Zn, ^78^Se, and ^80^Se. 

The concentrations were calculated using external calibration with internal standardization. The control material for the measurement of the brain solutions was each prepared in triplicate.

### 2.3. Statistics

Statistical analysis was performed using Graph Pad Prism Version 8.4.2 (464) (GraphPad Software, La Jolla, CA, USA), and values tested for significance using one-way ANOVA followed by Tukey post-hoc tests, or *t*-tests for pair-wise comparison. All values were normally distributed. In experiments using pooled samples or three replicates, normal distribution was not tested but assumed as the most likely scenario. “*n*” indicates biological replicates except for pig, where only one brain could be obtained and analytical replicates are reported. Statistical tests were two tailed with a significance level of α ≤ 0.05. Significances are stated with *p* values < 0.05 *; <0.01 **; <0.001 ***. Results are shown as mean and standard deviation. 

## 3. Results

### 3.1. Fixation of Tissue Alters Trace Metal Levels in Brain Tissue

**Figure 1:**
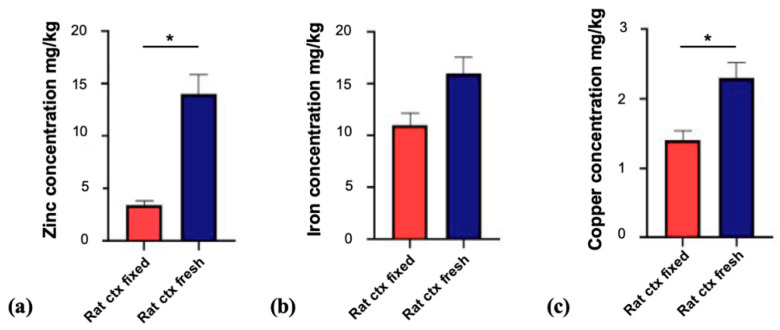
The concentrations of essential trace metals in the cerebral cortex (ctx) from rat using fixed and fresh frozen tissue. (**a**) The comparison of zinc concentrations within the fixed cortex vs. fresh Figure 3.4 ± 0.45 vs. 14 ± 1.9 mg/kg; *n* = 2; *p* = 0.0165); (**b**) The iron concentrations within the fixed cortex vs. fresh frozen cortex tissue revealed higher levels in fresh frozen tissue that are not statistically significant (16 ± 1.6 vs. 11 ± 1.2 mg/kg; *n* = 2; *p* = 0.0715); (**c**) A significantly higher copper concentration in fresh frozen cortex tissue (1.4 ± 0.14 vs. 2.3 ± 0.22 mg/kg; *n* = 2; *p* = 0.0395) were found. Significances are stated with *p* values < 0.05 *.

In the first set of experiments, we compared fresh frozen brain tissue (from rat) with tissue from a perfused (fixed in Carolina’s Perfect Solution, Carolina Biological, Burlington, NC, USA) animal, to investigate whether differences in the literature may result from the use of different protocols and methods of tissue preparation. This is particularly important, since almost all studies using human brain tissue are based on fixed tissue, while frequently, studies using mouse and rat as human model organism are based on fresh frozen brains. Our results show that the concentrations of the measured trace metals (zinc, iron, and copper) within the fresh frozen cerebral cortex of rats and fixed cortex are different. The concentrations obtained from fresh (snap frozen) cortex were found to be significantly higher for zinc and copper (*p* < 0.05) (Figure 1).

Based on these results, all further experiments were carried out on fresh frozen tissue without fixation or perfusion of the animal, following the same protocol for all sample preparations.

### 3.2. Trace Metal Levels in Brain Tissue of Vertebrates

**Figure 2:**
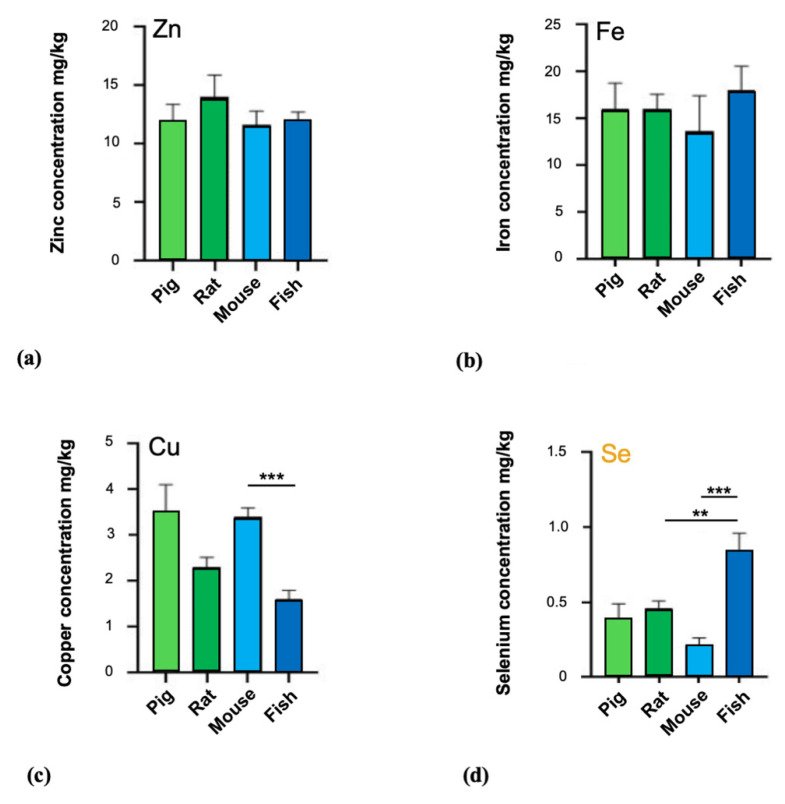
Concentrations of zinc, iron, copper, and selenium in fresh cortex brain tissues of the Vertebrates (Chordata). (**a**) The comparison of zinc concentrations within the brains of pig (12 ± 1.4 mg/kg; *n* = 2), rat (14 ± 1.9 mg/kg; *n* = 2), mouse (11.67 ± 1.155 mg/kg; *n* = 3), and fish (12 ± 0.76 mg/kg; *n* = 6) did not reveal any significant differences (Welch’s ANOVA test *p* = 0.7054). (**b**) Iron levels in brain tissue of pig (16 ± 2.8 mg/kg; *n* = 2), rat (16 ± 1.6 mg/kg; *n* = 2), mouse (13.67 ± 3.786 mg/kg; *n* = 3), and fish (18 ± 2.6 mg/kg; *n* = 6) were not significantly different between species (Welch’s ANOVA test *p* = 0.5312). (**c**) Significant differences were found in the distribution of copper levels: pig brain (3.5 ± 0.61 mg/kg; *n* = 2), rat (2.3 ± 0.22 mg/kg; *n* = 2), mouse (3.4 ± 0.2 mg/kg; *n* = 3), and fish (1.6 ± 0.2 mg/kg; *n* = 6), (Welch’s ANOVA test *p* = 0.0130). Post-hoc tests show a significant difference between mouse and fish (*p* = 0.0007). (**d**) Selenium levels in the brain of pig (0.38 ± 0.11 mg/kg; *n* = 2), rat (0.46 ± 0.048 mg/kg; *n* = 2), mouse (0.21 ± 0.05 mg/kg; *n* = 3), and in the brain of the fish (0.84 ± 0.12 mg/kg; *n* = 6) show significant differences (Welch’s ANOVA test *p* = 0.0093). Post-hoc reveal statistically relevant differences between rat and fish (*p* = 0.0050) and mouse and fish (*p* < 0.0001). Significances are stated with *p* values < 0.01 **; <0.001 ***.

In the next set of experiments, we assessed the concentration of iron, zinc, copper, and selenium in brain tissue (Cortex) of the vertebrates (Chordata): pig, rat, mouse, and fish (Figure 2). The data obtained did not show any significant differences between pig, rat, mouse, and fish regarding zinc and iron levels (*p* > 0.05). On the contrary, significant differences were found for copper and selenium levels. Significant differences for copper were observed in the multiple comparisons between mouse vs. fish (*p* = 0.0007). For selenium levels in brain, significant differences were detected between rodents and (marine) fish (rat and fish (*p* = 0.0050) and mouse and fish (*p* ≤ 0.0001)). 

### 3.3. Trace Metal Levels in Brain Tissue of Invertebrates

**Figure 3:**
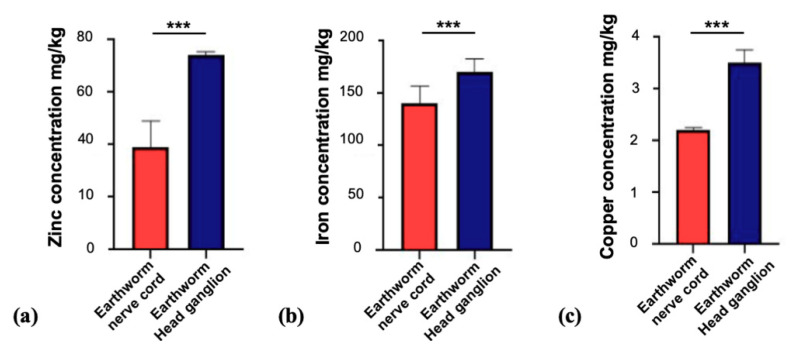
Concentrations of trace metals in the nervous system of the earthworm. (**a**) Zinc concentrations were higher in the head ganglion of earthworms (74 ± 1.4 mg/kg; *n* = 15) compared to the zinc levels in the nerve cord of the worms (39 ± 10 mg/kg; *n* = 15) (*t*-test, *p* < 0.0001). (**b**) Iron levels in the head ganglion (170 ± 13 mg/kg; *n* = 15) were significantly different compared to the nerve cord (140 ± 0.2 mg/kg; *n* = 15) (*t*-test, *p* < 0.0001). (**c**) Copper concentrations were significantly different between nerve cord (2.2 ± 0.054 mg/kg; *n* = 15) and earthworm head ganglion (3.5 ± 0.26 mg/kg; *n* = 15) (*t*-test, *p* < 0.0001). Significances are stated with *p* values < 0.001 ***.

Next, we investigated the trace metal and selenium levels in model species outside the phylum Chordata (subphylum Vertebrata). To that end, we analyzed the nervous system of the earthworm (Annelida), locusts (Arthropoda), and snails (Mollusca). Given that the brain of these animals is much less centralized and especially the earthworm contains a nerve cord that in each segment harbors ganglia that contain nerve cells, we first prepared the head ganglion of earthworms and the nerve cord with each ganglion in each segment of the worm separately. The results show that iron was the highest trace element measured in earthworms and copper the lowest in both head ganglion and nerve cord. The trace metals analyzed are significantly higher concentrated in the earthworm head ganglion (*p* < 0.0001). Zinc and iron were the most abundant trace metals analyzed in the “brain” (Figure 3). The levels of selenium were below the detection limit of 0.1 mg/kg.

**Figure 4:**
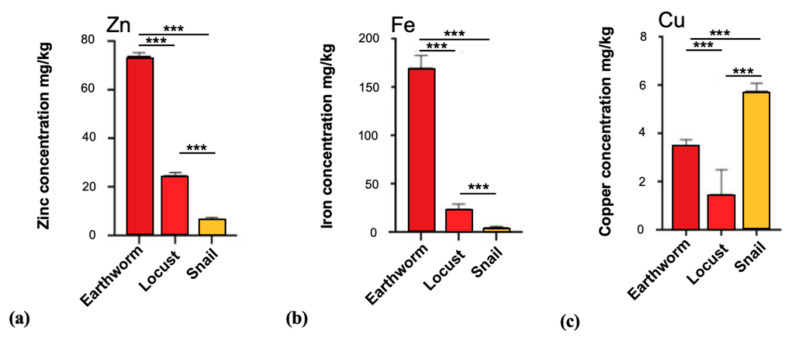
Concentrations of trace metals in earthworm, locust, and snail. (**a**) The comparison of zinc concentrations within the head ganglion of earthworm, locust, and snail resulted in statistically significant differences (Welch’s ANOVA test *p* < 0.0001). Post-hoc analysis: earthworm vs. locust (*p* < 0.0001), earthworm vs. snail (*p* < 0.0001), and locust vs. snail (*p* < 0.0001). (**b**) Iron levels were significantly different in earthworm, locust, and snail (Welch’s ANOVA test *p* < 0.0001). Post-hoc analysis: earthworm vs. locust (*p* < 0.0001), earthworm vs. snail (*p* < 0.0001), and locust vs. snail (*p* < 0.0001). (**c**) Copper levels in the “brain” of earthworm, locust, and snail are different (Welch’s ANOVA test *p* < 0.0001). Post-hoc analysis: earthworm vs. locust (*p* < 0.0001), earthworm vs. snail (*p* < 0.0001), and locust vs. snail (*p* < 0.0001). Significances are stated with *p* values < 0.001 ***.

Based on these results, for all animals (earthworm, locust, and snail), we focused on the head ganglion as the brain of the animals for our subsequent analyses. Our results show that, in the head ganglion of the earthworm, the most abundant trace metal is iron (170 ± 13 mg/kg; *n* = 15), followed by zinc (74 ± 1.4 mg/kg; *n* = 15) and copper (3.5 ± 0.26 mg/kg; *n* = 15). In locust samples, we found that the concentrations of zinc and iron were similar, while copper is represented with the lowest value between the three species: zinc (25 ± 1 mg/kg; *n* = 15), iron (24 ± 5.3 mg/kg; *n* = 15), and copper (1.5 ± 1 mg/kg; *n* = 15) respectively. Interestingly for snails, as for locusts, the trace metal with the highest concentration in the head ganglion is zinc (7 ± 0.65 mg/kg; *n* = 24), but in contrast to the other two species, copper concentrations are higher than iron concentrations: copper (5.8 ± 0.3 mg/kg; *n* = 24) and iron (5.4 ± 0.8 mg/kg; *n* = 24) (Figure 4). The levels of selenium were below the detection limit of 0.1 mg/kg in all species.

### 3.4. The Trace Element Levels in Brain Tissue Across All Phyla Indicate Active Species-Specific Regulation

Finally, we compared the trace metal levels in brain tissue across all phyla. In detail we analyzed the concentrations of zinc, iron, and copper in the nervous system of the vertebrates (Chordata): average of pig, rat, mouse, and fish, in Annelida (model organism: earthworm), in Arthropods (locust), and in Mollusca (snail). Although the analysis is limited by the use of only one model species for Annelida, Arthropods, and Mollusca, the results show that the zinc levels between vertebrates and invertebrates were statistically significant (*p* < 0.0001), with Annelida (74 ± 1.4 mg/kg; *n* = 15) showing the highest zinc level and Mollusca (7 ± 0.65 mg/kg; *n* = 24) the lowest; the values analyzed for the other two phyla are: Arthropoda (25 ± 1 mg/kg; *n* = 15) and Chordata (12.42 ± 1.07 mg/kg; *n* = 13). In addition to this, the statistical analysis performed on the samples confirms that the concentrations of iron across all phyla show significant differences (*p* < 0.0001). In detail, we found the highest value in earthworm (Annelida) (170 ± 13 mg/kg; *n* = 15), followed by locust (Arthropoda) (24 ± 5.3 mg/kg; *n* = 15), Chordata (15.92 ± 1.77 mg/kg; *n* = 13), and Mollusca with the least value (5.4 ± 0.8 mg/kg; *n* = 24). Significant differences were also found for copper in concentrations (*p* < 0.0001). The phylum with the highest concentration of copper was the Mollusca (snail) and the lowest concentration was found in locust (1.5 ± 1 mg/kg; *n* = 15). The values found for Chordata and earthworm were 2.7 ± 0.91 mg/kg (*n* = 13) and 3.5 ± 0.26 mg/kg (*n* = 15), respectively (Figure S1). 

### 3.5. The Trace Metals Levels in Brain Tissue Are Not Correlated with the Brain Weight 

**Figure 5:**
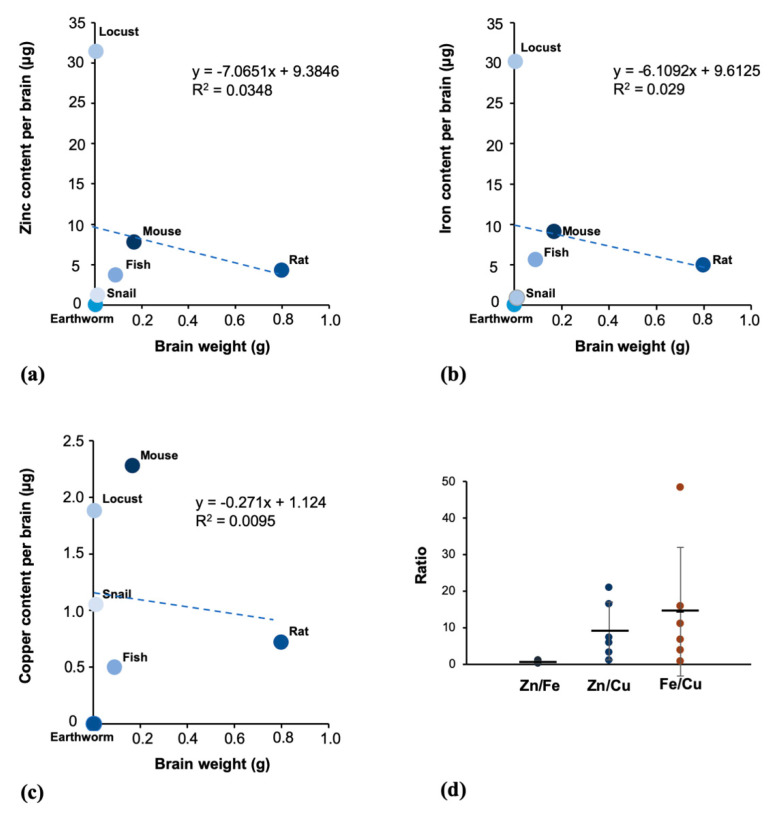
Correlation between total zinc (μg) (**a**), iron (**b**), and copper (**c**), and brain weight (g). A Pearson correlation analysis shows the following data: zinc: r = −0.1866, *p* = 0.7234, and the iron: r = −0.1704, *p* = 0.7469, copper: r = −0.0973, *p* = 0.8545. Thus, no significant correlation between brain weight and zinc, iron, and copper content has been found. (**d**) The ratio between zinc and iron (mg/kg brain weight) is relatively constant across all species (average 0.86 ± 0.3 SD), while the brain zinc/copper (9.34 ± 7.85 SD) and iron/copper (14.62 ± 17.46 SD) ratio is much more variable across different species.

We finally investigated the correlation between the total zinc, iron, and copper found in the brain of each species and the brain weight of the species analyzed (Figure 5a–c). With the results obtained we performed a correlation analysis (Pearson’s correlation). The results show that there is no correlation between zinc, iron, and copper concentration and the brain weight of the species. However, there is a relationship between zinc (Figure 5a) and iron (Figure 5b) content in the brain of each species, resulting in a relatively constant Zn/Fe ratio of around 0.86 across all species. In contrast, a much higher variation in the Zn/Cu and Fe/Cu ratios is found (Figure 5d).

## 4. Discussion

Zinc is one of the most abundant trace metals in the brain, playing a pivotal role in neuronal activities, synaptic plasticity, and in the regulation of post-synaptic proteins [57,58]. Its importance is underlined by the association of zinc deficiency with brain disorders such as ASD, ADHD, and depression [59,60]. Similarly, iron and copper are essential for a healthy brain function.

The aim of this study was to gain more insights on the physiological levels of zinc, copper, and iron in the brain tissue of various vertebrates and invertebrates. In addition, multiple studies have been conducted in the past analyzing the levels of trace metals in the brain. However, the reported trace metal concentrations show high variability. With this work we also wanted to investigate the reasons for conflicting information in the literature. 

In Table 1, we report the zinc levels in the brain tissue found in several studies. The data were obtained using AAS or ICP-MS, two of the most used, accepted, and reliable methods for the analysis of the trace metals. The use of AAS or ICP-MS seems not to result in consistently lower or higher values that can be associated with one of the technologies. Instead, it seems likely that the variability results from divergent tissue preparation methods. Our results, for example, confirm a significant influence of tissue fixation on trace metal content. One reason for the lower levels of trace metals measured after fixation may be the replacement of blood through perfusion of animals with fixation chemicals or bathing of tissue in fixation solution that depletes metals from disrupted cells. Therefore, our study is highly suited for a comparative analysis, as all brain samples have been prepared in the same manner and analyzed with the same technology.

We measured the levels of zinc, iron, copper, and selenium in samples belonging to the phylum of Chordata (Vertebrates) analyzing the cortical brain tissue of pig, rat, mouse, and fish. Our data are in line, and therefore confirms the reported concentrations of these metals, in particular of zinc, found in the literature (Table 2). Thus, pig, mice, rats, and fish seem to share an average zinc concentration of around 15 mg/kg in cortical tissue. This is in line with the concentration of zinc in human brain tissue that averages also around 19 mg/kg [10,46,47,48,49,50]. Thus, rodents are indeed a suitable model system to study human trace metal biology in the brain.

Despite similar zinc (and iron) levels, significant differences have been detected for copper and selenium, where fish diverge from the mammalian species by showing lower copper but higher selenium levels in the brain. Elevated levels of selenium have been reported in fish before [61] and may be associated with the presence of a higher number of selenoproteins in fish compared to mammals [62].

In the present study we also measured the trace metal levels in various invertebrates species. The results showed significant differences in zinc, copper, and iron levels between the earthworm head ganglion and its nerve cord. The trace metals levels were higher in the head ganglion than in the nerve cord. Thus, although there is a lack of a blood-brain barrier (BBB) in earthworms (and also in insects) active transport mechanisms must exist that enrich specific trace metals in the head ganglion [63].

Similarly, insects are devoid of an arterial and venous system across the neural mass. They have hemolymph, able to bath the outer surfaces of ganglia and nerves. In addition to this, insects present a thin layer made by perineural and glial cells that covers the nerves, creating in this way a barrier representing a challenge for the crossing of ions [64]. Thus, also in insects, transport mechanisms need to mediate the enrichment of trace metals in the insect brain.

Here, we compared the levels of zinc, iron, and copper in the head ganglions/brains of earthworm, snail, and locust. The results reveal significant differences across the phyla. The snail brain shows high levels of copper (higher than iron and zinc) in line with published data (Table 2). This can be explained by the different way Mollusca transport oxygen. Instead of iron that binds oxygen in Chordata and the earthworm, in snails, the transport of oxygen occurs through a compound known as hemocyanin, a molecule that presents an atom of copper (instead of iron) that binds oxygen [65]. However, the high copper concentrations may complicate the enrichment of metals such as iron and zinc. Especially zinc and copper have an antagonistic relationship because of similarities in zinc and copper binding sites on proteins and a resulting competition [66]. In line with this, we have measured the lowest zinc and iron levels of all analyzed species in the snail brain. The earthworm head ganglion, in contrast, contained remarkably high levels of iron and zinc.

The comparison of the iron, zinc, and copper levels measured in this study with published values for earthworm, locust, and snail show significant differences. Interestingly, previous studies report even higher levels of iron and zinc in the nervous system of the earthworm and locust (Table 2). However, in line with the literature, earthworm, and locust brains were also in our comparisons the nervous tissues with highest iron and zinc levels, confirming the high abundance of these metals in these species, whose specific function remains mostly unknown.

The significant differences may be attributed to the different preparation protocols used for the analysis, or to the different ages and species used. The environment/nutrition of the animals may play a minor role as our results hint at an active regulation of trace metal content in the nervous tissue of the animals. Of note is the high standard deviation (e.g., for human or earthworm) hinting at high variation between individuals/studies in some species. This could be due to alternative sample collection and preparation, or if subsamples of the brain were taken that show heterogeneity in metal distribution [67].

In the last analysis, we investigated a correlation between metal levels found in each brain of a species and its brain weight. If the function of trace metals in the brain is similar in all species, a correlation is expected with larger brains containing larger quantities of the metal, irrespective of other factors such as cognitive performance. However, we could not establish a significant correlation between brain-weight and the levels of the essential trace metals. Therefore, the regulation and utilization of specific trace metals may be an important factor that determines specific functions of the animal/human nervous system.

## 5. Conclusions

Trace metals homeostasis is essential for the healthy development and functioning of the brain. Despite the known specific accumulation of metals, i.e., zinc, in the hippocampus of mammals, zinc, and also iron and copper are found in the nervous system outside the class of Mammalia, across all phyla, in Annelida, Arthropoda, Mollusca, and Chordata. In different species representing these phyla, significantly different trace metals levels can be observed, hinting at the presence of specific regulatory mechanisms that already exist in very rudimentary nervous systems to enrich certain trace metals in specific regions within the nervous tissue. The function of this controlled trace metal distribution, however, is mostly unknown outside the mammalian brain. Knowing that the metallome plays a key functional role in the nervous system from early on underlines the finding that controlled manipulation of trace metal homeostasis in the brain can represent a so far under-explored strategy for the treatment of neurological diseases [73].

## Figures and Tables

**Table 1:** Original research reporting zinc, iron and copper concentrations in brain tissue of different species. AAS: atomic absorption spectrophotometry; ICP-MS: inductively coupled plasma mass spectrometry; LA: laser ablation; INAA: Instrumenatal neutron activation analysis.

Reference	Species	Brain or Brain Region- Zinc mg/kg ^1^	Brain or Brain Region- Iron mg/kg ^1^	Brain or Brain Region- Copper mg/kg ^1^	Method
[36]	Wistar Rat	Cortex 23.30 ± 0.91	Cortex 28.87 ± 0.11	Cortex 2.20 ± 0.06	AAS
		Striatum 25.34 ± 0.67	Striatum. 25.14 ± 0.07	Striatum 2.18 ± 0.07	
		Cerebellum 32.93 ± 0.12	Cerebellum 30.13 ± 0.16	Cerebellum 2.69 ± 0.08	
[37]	Wistar Rat	Cerebellum 15.00 ± 5.50	-	-	ICP-MS
[38]	Albino Rat	Cortex 11.67 ± 1.04	-	-	AAS
		Hippocamp. 12.33 ± 2.75	-	-	
		Cerebellum 10.50 ± 3.78	-	-	
[39]	Wistar Rat	Whole brain 3.13 ± 0.07	Whole brain 2.81 ± 0.10	Whole Brain 1.79 ± 0.21	AAS
[40]	Marmoset	Cortex 15.91	Cortex 26.73	-	LA-ICP-MS
		Hippocampus 13.53	Hippocampus 20.77	-	
		Thalamus 14.62	Thalamus 27.52	-	
[41]	Mouse	Whole brain 10−15	-	-	LA-ICP-MS
[42]	Mouse	Hippocampus 30	-	-	AAS
		Cortex 16	-	-	
		Whole brain 18	-	-	
[43]	Mouse	Hippocampus > 4	Hippocampus 2–38	Hippocampus 0–1	LA-ICP-MS
[44]	Mouse	Cortex 8.3 ± 1.3	Cortex 3.5 ± 0.2	Cortex 1.7 ± 0.2	LA-ICP-MS
		Sub. Nigra 6.6 ± 1.5	Sub. Nigra 4.2 ± 0.7	Sub. Nigra 1.7 ± 0.3	
		Striatum 7.3 ± 0.5	Striatum 3.7 ± 0.5	Striatum 1.6 ± 0.1	
[45]	Lamb	Prefrontal cort. 4.03 ± 0.37	Prefrontal cort. 34.17 ± 1.43	Prefrontal cort16.83 ± 0.82	AAS
		Hippocamp. 2.72 ± 0.30	Hippocamp. 13.77 ± 0.72	Hippocamp. 17.07 ± 0.55	
		Thalamus 2.41 ± 0.25	Thalamus 17.38 ± 0.68	Thalamus 9.83 ± 0.51	
[46]	Human	Thalamus 12.3 ± 3.23	Thalamus 49.0 ± 10.9	Thalamus 4.83 ± 1.53	ICP-MS
		Sub. Nigra 14.6 ± 5.20	Sub. Nigra 210.3 ± 74.6	Sub. Nigra 16.45 ± 6.13	
		Frontal cortex 12.2 ± 3.48	Frontal cortex 27.4 ± 8.7	Frontal cortex 3.94 ± 0.99	
		Basal ganglia 12.3 ± 2.67	Basal ganglia 147.1 ± 49.4	Basal ganglia 6.50 ± 1.61	
[47]	Human	Hippocampus 9.63 ± 0.47	Hippocampus 3.38 ± 0.19	-	INAA
[48]	Human	Hippocampus 10	-	Hippocampus 14	LA-ICP-MS
[49]	Human	Hippocampus 6.5 ± 0.5	Hippocampus 26.6 ± 7.6	Hippocampus 4.0 ± 1.0	ICP-AES
		Cerebellum 8 ± 1	Cerebellum 45.4 ± 19.3	Cerebellum 7.4 ± 2.5	
[50]	Human	Hippocampus 64 ± 5–15	Hippocampus 342 ± 5–15	Hippocampus 65 ± 5–15	ICP-AES
[10]	Human	Frontal cortex 62 ± 12	-	Frontal cortex 21 ± 7	ICP-MS
		Hippocampus 70 ± 10	-	Hippocampus 19 ± 9	
		Cerebellum 36 ± 8	-	Cerebellum 22 ± 7	

^1^ All concentration given in the studies have been converted to mg/kg brain tissue to facilitate comparisons.

**Table 2:** Comparison of the average value of published trace metal levels with the results of this study. Zinc in mg/kg brain weight.

Species	Zn in Literature	Zn in This Study	Zn *p*-Value	Fe in Literature	Fe in This Study	Fe *p*-Value	Cu in Literature	Cu in This Study	Cu *p*-Value
Human [10,46,47,48,49,50]	19.5 ± 21.30	-	-	14.99 ± 16.42	-	-	12.33 ± 7.64	-	-
Pig [68]	9.24 ± 1.70	12 ± 1.4	<0.0001	58.78 ± 0.4	16 ± 2.8	<0.0001	0.306 ± 1.51	3.5 ± 0.61	0.0315
Mouse [41,42,44]	15.25 ± 3.89	12 ± 1.2	0.2456	3.8 ± 0.36	13.67 ± 3.79	<0.0001	1.67 ± 0.06	3.4 ± 0.2	<0.0001
Rat [36,37,38,39]	17.49 ± 8.22	14 ± 1.9	0.5717	21.74 ± 12.80	16 ± 1.6	0.5615	2.22 ± 0.37	2.3 ± 0.22	0.7707
Fish [69]	12.76 ± 4.62	12 ± 0.76	0.6993	-	18 ± 2.6	-	-	1.6 ± 0.2	-
Snail [70]	19.81 ± 5.14	7 ± 0.65	<0.0001	-	5.4 ± 0.8	-	14.8 ± 10.1	5.8 ± 0.3	<0.0001
Locust [71]	89.0 ± 3.0	25 ± 1	<0.0001	65 ± 2.0	24 ± 5.3	<0.0001	16.3 ± 0.7	1.5 ± 1	<0.0001
Earthworm [72]	284.0 ± 111.4	74 ± 1.4	<0.0001	1383 ± 577.6	170 ± 13	<0.0001	10.08 ± 1.429	3.5 ± 0.26	<0.0001

## Supplementary Materials

The following are available online at https://www.mdpi.com/2076-3425/10/7/460/s1, Table S1: Regression analysis between MEP_test_/Mmax ratio (independent variable) and SICI_Mmax_ (dependent variable) estimated individually for each subject with TS intensity set at 120%MT (left) and 130%MT (right). Figure S1. Trace metal levels analyzed in the brain tissues of animals belonging to the phyla Chordata, Annelida, Arthropoda and Mollusca.

## References

[1] World Health Organization (1996). Trace Elements in Human Nutrition and Health.

[2] Mehri A. (2020). Trace Elements in Human Nutrition (II)—An Update. Int. J. Prev. Med..

[3] Kobayashi M., Shimizu S. (1999). Cobalt proteins. Eur. J. Biochem..

[4] Warren M.J., Raux E., Schubert H.L., Escalante-Semerena J.C. (2002). The biosynthesis of adenosylcobalamin (vitamin B12). Nat. Prod. Rep..

[5] Banerjee R., Ragsdale S.W. (2003). The many faces of vitamin B12: Catalysis by cobalamin-dependent Enzymes. Annu. Rev. Biochem..

[6] De Renzo E.C., Heytler P.G., Kaleita E. (1954). Further evidence that molybdenum is a co-factor of xanthine oxidase. Arch. Biochem. Biophys..

[7] Mahler H.R., Mackler B., Green D.E., Bock R.M. (1954). Studies on metalloflavoproteins III. Aldehyde oxidase: A molybdoflavoprotein. J. Biol. Chem..

[8] Nicholas D.J., Nason A., McLeroy W.D. (1954). Molybdenum and nitrate reductase. Ind. J. Biol. Chem..

[9] Ramos P., Santos A., Pinto N.R., Mendes R., Magalhães T., Almeida A. (2014). Iron levels in the human brain: A post-mortem study of anatomical region differences and age-related changes. J. Trace Elem. Med. Biol..

[10] Ramos P., Santos A., Pinto N.R., Mendes R., Magalhães T., Almeida A. (2014). Anatomical region differences and age-related changes in copper, zinc, and manganese levels in the human brain. Biol. Trace Elem. Res..

[11] Kambe T., Tsuji T., Hashimoto A., Itsumura N. (2015). The Physiological, Biochemical, and Molecular Roles of Zinc Transporters in Zinc Homeostasis and Metabolism. Physiol. Rev..

[12] Kardos J., Héja L., Simon Á., Jablonkai I., Kovács R., Jemnitz K. (2018). Copper signalling: Causes and consequences. Cell Commun. Signal..

[13] Yehuda S., Youdim M.B. (1989). Brain iron: A lesson from animal models. Am. J. Clin. Nutr..

[14] Eisenstein R.S. (2000). Iron regulatory proteins and the molecular control of mammalian iron metabolism. Annu. Rev. Nutr..

[15] Wigglesworth J.M. (1998). Iron dependent enzymes in the brain. Brain Iron: Neurochemical and Behavioral Aspects.

[16] Kwik-Uribe C.L., Gietzen D., German J.B., Golub M.S., Keen C.L. (2000). Chronic marginal iron intakes during early development in mice result in persistent changes in dopamine metabolism and myelin composition. J. Nutr..

[17] Larkin E.C., Ananda Rao G., Dobbing J. (1990). Importance of fetal and neonatal iron: Adequacy for normal development of central nervous system. Brain, Behaviour, and Iron in the Infant Diet.

[18] Clardy S.L., Wang X., Zhao W., Liu W., Chase G.A., Beard J.L., Felt B.T., Connor J.R., Parvez H., Riederer P. (2006). Acute and chronic effects of developmental iron deficiency on mRNA expression patterns in the brain. Oxidative Stress and Neuroprotection.

[19] Dallman P.R. (1986). Biochemical basis for the manifestations of iron deficiency. Annu. Rev. Nutr..

[20] Georgieff M.K. (2008). The role of iron in neurodevelopment: Fetal iron deficiency and the developing hippocampus. Biochem. Soc. Trans..

[21] Bredholt M., Frederiksen J.L. (2016). Zinc in multiple sclerosis: A systematic review and meta-analysis. ASN Neuro.

[22] Tapiero H., Tew K.D. (2003). Trace elements in human physiology and pathology: Zinc and metallothioneins. Biomed. Pharmacother..

[23] Prasad A.S. (2008). Clinical, immunological, anti-inflammatory and antioxidant roles of zinc. Exp. Gerontol..

[24] Levenson C.W., Morris D. (2011). Zinc and neurogenesis: Making new neurons from development to adulthood. Adv. Nutr..

[25] Fukada T., Yamasaki S., Nishida K., Murakami M., Hirano T. (2011). Zinc homeostasis and signaling in health and diseases. JBIC J. Biol. Inorg. Chem..

[26] Takeda A. (2001). Zinc homeostasis and functions of zinc in the brain. Biometals.

[27] Prasad A.S. (2009). Impact of the discovery of human zinc deficiency on health. J. Am. Coll. Nutr..

[28] DiGirolamo A.M., Ramirez-Zea M. (2009). Role of zinc in maternal and child mental health. Am. J. Clin. Nutr..

[29] Peña M.M., Lee J., Thiele D.J. (1999). A delicate balance: Homeostatic control of copper uptake and distribution. J. Nutr..

[30] Rucker R.B., Kosonen T., Clegg M.S., Mitchell A.E., Rucker B.R., Uriu-Hare J.Y., Keen C.L. (1998). Copper, lysyl oxidase, and extracellular matrix protein cross-linking. Am. J. Clin. Nutr..

[31] Kaler S.G. (1998). Diagnosis and therapy of Menkes syndrome, a genetic form of copper deficiency. Am. J. Clin. Nutr..

[32] Kodama H., Fujisawa C. (2009). Copper metabolism and inherited copper transport disorders: Molecular mechanisms, screening, and treatment. Metallomics.

[33] Yasuda H., Yoshida K., Yasuda Y., Tsutsui T. (2011). Infantile zinc deficiency: Association with autism spectrum disorders. Sci. Rep..

[34] Grabrucker A.M. (2013). Environmental factors in autism. Front. Psychiatry.

[35] Bjørklund G. (2013). The role of zinc and copper in autism spectrum disorders. Acta Neurobiol. Exp. (Wars).

[36] Pal A., Prasad R. (2016). Regional distribution of copper, zinc and iron in brain of Wistar rat model for non-Wilsonian brain copper toxicosis. Indian J. Clin. Biochem..

[37] Yakimoskii A.F., Shantyr I.I., Vlasenko M.A., Yakovleva M.V. (2017). Effects of Acyzol on Zinc Content in Rat Brain and Blood Plasma. Bull. Exp. Biol. Med..

[38] Takeda A., Takefuta S., Okada S., Oku N. (2000). Relationship between brain zinc and transient learning impairment of adult rats fed zinc-deficient diet. Brain Res..

[39] Fayed A.H.A. (2010). Brain trace element concentration of rats treated with the plant alkaloid, vincamine. Biol. Trace Elem. Res..

[40] Knauer B., Majka P., Watkins K.J., Taylor A.W.R., Malamanova D., Paul B., Yu H.H., Bush A.I., Hare D.J., Reser D.H. (2017). Whole-brain metallomic analysis of the common marmoset (Callithrix jacchus). Metallomics.

[41] Portbury S.D., Hare D.J., Bishop D.P., Finkelstein D.I., Doble P.A., Adlard P.A. (2018). Trehalose elevates brain zinc levels following controlled cortical impact in a mouse model of traumatic brain injury. Metallomics.

[42] Yang Y., Jing X.P., Zhang S.P., Gu R.X., Tang F.X., Wang X.L., Xiong Y., Qiu M., Sun X.Y., Ke D. (2013). High dose zinc supplementation induces hippocampal zinc deficiency and memory impairment with inhibition of BDNF signaling. PLoS ONE.

[43] Paul B., Hare D.J., Bishop D.P., Paton C., Cole N., Niedwiecki M.M., Andreozzi E., Vais A., Billings J.L., Bray L. (2015). Visualising mouse neuroanatomy and function by metal distribution using laser ablation-inductively coupled plasma-mass spectrometry imaging. Chem. Sci..

[44] Matusch A., Depboylu C., Palm C., Wu B., Höglinger G.U., Schäfer M.K.H., Becker J.S. (2010). Cerebral bioimaging of Cu, Fe, Zn, and Mn in the MPTP mouse model of Parkinson’s disease using laser ablation inductively coupled plasma mass spectrometry (LA-ICP-MS). J. Am. Soc. Mass Spectrom..

[45] Bakirdere S., Kizilkan N., Yaman M. (2011). Determination of zinc, copper, iron, and manganese in different regions of lamb brain. Biol. Trace Elem. Res..

[46] Krebs N., Langkammer C., Goessler W., Ropele S., Fazekas F., Yen K., Scheurer E. (2014). Assessment of trace elements in human brain using inductively coupled plasma mass spectrometry. J. Trace Elem. Med. Biol..

[47] Höck A., Demmel U., Schicha H., Kasperek K., Feinendegen L.E. (1975). Trace element concentration in human brain: Activation analysis of cobalt, iron, rubidium, selenium, zinc, chromium, silver, cesium, antimony and scandium. Brain.

[48] Becker J.S., Zoriy M.V., Pickhardt C., Palomero-Gallagher N., Zilles K. (2005). Imaging of copper, zinc, and other elements in thin section of human brain samples (hippocampus) by laser ablation inductively coupled plasma mass spectrometry. Anal. Chem..

[49] Rajan M.T., Rao K.J., Mamatha B.M., Rao R.V., Shanmugavelu P., Menon R.B., Pavithran M.V. (1997). Quantification of trace elements in normal human brain by inductively coupled plasma atomic emission spectrometry. J. Neurol. Sci..

[50] Andrasi E., Orosz L., Bezur L., Ernyei L., Molnár Z. (1995). Normal human brain analysis. Microchem. J..

[51] Vilella A., Belletti D., Sauer A.K., Hagmeyer S., Sarowar T., Masoni M., Stasiak N., Mulvihill J.J., Ruozi B., Forni F. (2018). Reduced plaque size and inflammation in the APP23 mouse model for Alzheimer’s disease after chronic application of polymeric nanoparticles for CNS targeted zinc delivery. J. Trace Elem. Med. Biol..

[52] Corona C., Masciopinto F., Silvestri E., Del Viscovo A., Lattanzio R., La Sorda R., Ciavardelli D., Goglia F., Piantelli M., Canzoniero L.M.T. (2010). Dietary zinc supplementation of 3xTg-AD mice increases BDNF levels and prevents cognitive deficits as well as mitochondrial dysfunction. Cell Death Dis..

[53] Fourie C., Vyas Y., Lee K., Jung Y., Garner C.C., Montgomery J.M. (2018). Dietary Zinc Supplementation Prevents Autism Related Behaviors and Striatal Synaptic Dysfunction in Shank3 Exon 13–16 Mutant Mice. Front. Cell. Neurosci..

[54] Cezar L.C., Kirsten T.B., da Fonseca C.C.N., de Lima A.P.N., Bernardi M.M., Felicio L.F. (2018). Zinc as a therapy in a rat model of autism prenatally induced by valproic acid. Prog. Neuro Psychopharmacol. Biol. Psychiatry.

[55] Kirsten T.B., Queiroz-Hazarbassanov N., Bernardi M.M., Felicio L.F. (2015). Prenatal zinc prevents communication impairments and BDNF disturbance in a rat model of autism induced by prenatal lipopolysaccharide exposure. Life Sci..

[56] Rayman M.P. (2012). Selenium and human health. Lancet.

[57] Xie X., Smart T.G. (1994). Modulation of long-term potentiation in rat hippocampal pyramidal neurons by zinc. Pflügers Arch..

[58] Pfaender S., Grabrucker A.M. (2014). Characterization of biometal profiles in neurological disorders. Metallomics.

[59] Sauer A.K., Hagmeyer S., Grabrucker A.M., Erekolu P. (2016). Zinc Deficiency. Nutritional Deficiency.

[60] Petrilli M.A., Kranz T.M., Kleinhaus K., Joe P., Getz M., Johnson P., Malaspina D. (2017). The emerging role for zinc in depression and psychosis. Front. Pharmacol..

[61] Yoshida S., Haratake M., Fuchigami T., Nakayama M. (2011). Selenium in Seafood Materials. J. Health Sci..

[62] Lobanov A.V., Hatfield D.L., Gladyshev V.N. (2009). Eukaryotic selenoproteins and selenoproteomes. Biochim. Biophys. Acta.

[63] Stürzenbaum S.R., Kille P., Morgan A.J. (1998). The identification, cloning and characterization of earthworm metallothionein. FEBS Lett..

[64] Carlson S.D., Juang J.L., Hilgers S.L., Garment M.B. (2000). Blood barriers of the insect. Annu. Rev. Entomol..

[65] Van Holde K.E., Miller K.I., Decker H. (2001). Hemocyanins and invertebrate evolution. J. Biol. Chem..

[66] Keen C.L., Reinstein N.H., Goudey-Lefevre J., Lefevre M., Lönnerdal B., Schneeman B.O., Hurley L.S. (1985). Effect of dietary copper and zinc levels on tissue copper, zinc, and iron in male rats. Biol. Trace Elem. Res..

[67] Urgast D.S., Hill S., Kwun I.S., Beattie J.H., Goenaga-Infante H., Feldmann J. (2012). Zinc isotope ratio imaging of rat brain thin sections from stable isotope tracer studies by LA-MC-ICP-MS. Metallomics.

[68] Panayi A.E., Spyrou N.M., Ubertalli L.C., White M.A., Part P. (1999). Determination of trace elements in porcine brain by inductively coupled plasma-mass spectrometry, electrothermal atomic absorption spectrometry, and instrumental neutron activation analysis. Biol. Trace Elem. Res..

[69] Carpene E., Cattani O., Serrazanetti G.P., Fedrizzi G., Cortesi P. (1990). Zinc and copper in fish from natural waters and rearing ponds in Northern Italy. J. Fish Biol..

[70] Drozd Ł., Ziomek M., Szkucik K., Paszkiewicz W., Maćkowiak-Dryka M., Bełkot Z., Gondek M. (2017). Selenium, copper, and zinc concentrations in the raw and processed meat of edible land snails harvested in Poland. J. Vet. Res..

[71] Augustyniak M., Juchimiuk J., Przybyłowicz W.J., Mesjasz-Przybyłowicz J., Babczyńska A., Migula P. (2006). Zinc-induced DNA damage and the distribution of metals in the brain of grasshoppers by the comet assay and micro-PIXE. Comp. Biochem. Physiol. Part C Toxicol. Pharmacol..

[72] Zheng J., Goessler W., Geiszinger A., Kosmus W., Chen B., Zhuang G., Sui G. (1997). Multi-element determination in earthworms with instrumental neutron activation analysis and inductively coupled plasma mass spectrometry: A comparison. J. Radioanal. Nucl. Chem..

[73] Grabrucker A.M., Rowan M., Garner C.C. (2011). Brain-delivery of zinc-ions as potential treatment for neurological diseases: Mini review. Drug Deliv. Lett..

