# Supplementary material for: Concentrations of Essential Trace Metals in the Brain of Animal Species—A Comparative Study
Source: Brain Sci. 2020 Jul 17;10(7):460. doi: 10.3390/brainsci10070460 (PMC7407190; doi:10.3390/brainsci10070460)
Supplement: Supplementary file 1 [file brainsci-10-00460-s001.pdf]

brain sciences

MDPI

Supplementary material

Table S1. Method accuracy and precision checked by using the reference materials and comparing the results from the measurement with the certified values.

(a) Reference Whole Blood Level 1 (*n* = 8).

Element	Measured	Certified
	mg/kg	mg/kg
Zinc	5.0 ± 0.79	3.4–5.2
Iron	441 ± 42	350 (indicative)
Copper	0.65 ± 0.061	0.51–0.76
Selenium	0.062 ± 0.028	0.048–0.072

(b) Reference Serum Level 1 (*n* = 5).

Element	Measured	Certified
	mg/kg	mg/kg
Zinc	1.6 ± 0.24	0.95–1.24
Iron	1.2 ± 0.72	1.17-1.77
Copper	1.0 ± 0.053	0.99–1.18
Selenium	0.070 ± 0.0030	0.076–0.099

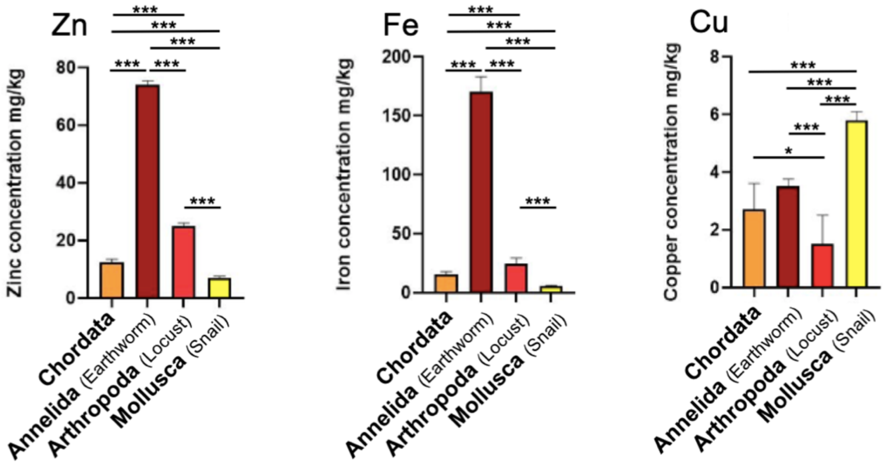

(a) (b) (c)

Figure S1. Trace metal levels analyzed in the brain tissues of animals belonging to the phyla Chordata, Annelida, Arthropoda and Mollusca. (**a**) Comparisons in zinc levels between Vertebrates and Invertebrates showed significant differences (Welch's ANOVA test $p < 0.0001$). A Post-hoc test reveals that Chordata have significantly lower zinc levels in brain tissue when compared to a member of the phylum Annelida ($p < 0.0001$) and Arthropoda ($p < 0.0001$). However, the levels found in Chordata brain tissue are significantly higher than those in Mollusca (snail) ($p < 0.0001$). (**b**) Iron levels are significantly different across members of the analyzed animal phyla (Welch's ANOVA test $p < 0.0001$). Post-hoc analysis: Chordata vs. Annelida (earthworm) ($p < 0.0001$), Chordata vs. Arthropoda (locust) ($p = 0.0002$), Chordata vs. Mollusca (snail) ($p < 0.0001$). As for zinc, Chordata have lower levels of iron than the members of the phyla Annelida and Arthropoda, but higher levels than Mollusca. (**c**) Copper levels are also significantly different between members of the phyla (Welch's ANOVA test $p < 0.0001$). Post-hoc analysis reveals: Chordata vs. Arthropoda (locust) ($p = 0.0164$), Chordata vs. Mollusca (snail) ($p < 0.0001$). Thus, Chordata (averaged from pig, rat, mouse, fish) have higher copper levels than a member of the phylum Arthropoda (locust) but lower levels when compared to Mollusca (snail). Significances are stated with p values < 0.05 *; < 0.001 ***.
